# Color matching and color recovery in large composite restorations using single-shade or universal composites

**DOI:** 10.1590/0103-6440202405665

**Published:** 2024-03-22

**Authors:** Ellen Dionélia Alencar Rodrigues Rosa, Larissa Fernandes Vieira Da Silva, Paula Fernanda Damasceno Silva, André Luís Faria e Silva

**Affiliations:** 1 Department of Dentistry, Federal University of Sergipe, Aracaju, SE, Brazil; 2 Graduate Program in Dentistry, Federal University of Sergipe, Aracaju, SE, Brazil

**Keywords:** Color assessments, Color blending, Single-shade composite, Resin composite

## Abstract

This study assessed the color-matching ability and color recovery of unprepared teeth when using single-shade composites and a universal composite in large restorations. Buccal and palatine surface colors of molars were measured with a spectrophotometer (CIELAB) before preparing round cavities (6 mm in diameter, 2 mm in depth). The cavities were randomly filled with a single-shade composite (Omnichroma, Diamond One, or Vittra APS Unique) or a universal composite (Filtek Universal). Color measurements of the restored cavities were taken, and overall color differences (ΔE_ab_ and ΔE_00_) and differences in the whitening index for dentistry (ΔWI_D_) from baseline were calculated. Additionally, visual assessments of a color match to the surrounding enamel were performed by forty evaluators (laypersons and undergraduate students of dentistry) in a viewing booth under illuminant D65, with rating scores from 0 (no color mismatch) to 4 (not acceptable). Data were analyzed using RM or one-way ANOVA (α = 0.05). Results showed that the restorations generally exhibited whiter colors (WI_D_ ranged from 27.9 to 41.3) than the unprepared teeth (WI_D_ ranged from 15.9 to 19.3). The composite Filtek Universal demonstrated the lowest color discrepancy (ΔWI_D_ = 8.6; ΔE_00_ = 10.8; and ΔE_00_ = 6.2), and no significant differences were observed among the evaluated single-shade composites. Furthermore, all composites showed similar and adequate color matches to the surrounding enamel. However, it is important to note that despite their ability to match the surrounding enamel reasonably, none of the composites evaluated in large restorations fully recovered the color observed in unprepared teeth.

## Resumo

Este estudo avaliou a habilidade de combinação de cores e recuperação de cor de dentes não preparados ao utilizar resinas compostas de única tonalidade e uma resina composta universal em grandes restaurações. As cores das superfícies bucais e palatinas de molares foram medidas com um espectrofotômetro (CIELAB) antes de preparar cavidades esféricas (6 mm de diâmetro, 2 mm de profundidade). As cavidades foram preenchidas aleatoriamente com uma resina composta de única tonalidade (Omnichroma, Diamond One ou Vittra APS Unique) ou com uma resina composta universal (Filtek Universal). Foram realizadas medições das cores das cavidades restauradas e calculadas as diferenças de cor globais (ΔE_ab_ and ΔE_00_)) e as diferenças no índice de clareamento para odontologia (ΔWI_D_) em relação à cor inicial. Além disso, foram realizadas avaliações visuais da correspondência de cor com o esmalte circundante por quarenta avaliadores (leigos e estudantes de odontologia) em uma cabine de visualização sob iluminante D65, com pontuações de 0 (nenhuma discrepância de cor) a 4 (não aceitável). Os dados foram analisados usando RM ou ANOVA unidirecional (α = 0,05). Os resultados mostraram que as restaurações geralmente apresentavam cores mais brancas (WI_D_ variou de 27,9 a 41,3) do que os dentes não preparados (WI_D_ variou de 15,9 a 19,3). A resina composta Filtek Universal demonstrou a menor discrepância de cor (ΔWI_D_ = 8,6; ΔE_00_ = 10,8; and ΔE_00_ = 6,2), e não foram observadas diferenças significativas entre as resinas compostas de única tonalidade avaliadas. Além disso, todas as resinas compostas apresentaram correspondência de cor similar e adequada com o esmalte circundante. No entanto, é importante observar que, apesar de sua capacidade de corresponder razoavelmente com o esmalte circundante, nenhuma das resinas compostas avaliadas em grandes restaurações recuperou completamente a cor observada nos dentes não preparados.

## Introduction

The stratification of composite resin restorations in aesthetic areas poses a challenge for clinicians since achieving color similarity between the tooth and the dental structure is often unpredictable ^1^ ^,^ ^2^. The determination of composite shade depends on factors such as proper illumination conditions, the distance between the observer and the substrate, as well as the clinician's experience, visual accuracy, fatigue, and mood, among others ^3^ ^,^ ^4^ ^,^ ^5^. Furthermore, the relationship between translucency and the composite resin's thickness strongly influences the restoration's final color ^6^ ^,^ ^7^. Using a more translucent composite or a thinner increment increases the visualization of the adjacent substrate, affecting the restoration's color ^8^.

Simplified composite systems have been developed to address these challenges, reduce technical sensitivity, and improve predictability. Universal composites offer a single translucency that can adapt to different clinical scenarios ^9^ ^,^ ^10^. However, they come in various shades, requiring clinicians to determine the most suitable match for the tooth color. In contrast, single-shade composites aim to streamline restorative procedures by eliminating the color selection step (11-18). These materials exhibit improved color adjustment potential, enabling them to mimic the underlying and surrounding substrates ^11^ ^,^ ^12^ ^,^ ^14^ ^,^ ^18^. Enhanced color adjustment is primarily attributed to the high translucency achieved after composite polymerization, resulting in a mirror effect of the underlying dental substrate. The increase in the resin matrix's refractive index during polymerization brings it closer to that of the inorganic filler, enabling efficient light transmission ^12^ ^,^ ^19^.

Numerous studies have evaluated simplified composite systems using artificial substrates to measure color adjustment ^11^ ^,^ ^12^ ^,^ ^14^ ^,^ ^15^ ^,^ ^16^ ^,^ ^18^. However, these studies have not considered the optical complexity of dental substrates. Additionally, when tooth substrates are used, the color match of the composite to the surrounding enamel is usually the only aspect assessed. Yet, in aesthetic areas, like with large direct composite restorations such as veneers, the restoration should match the colors of adjacent teeth, not just the surrounding enamel ^13^ ^,^ ^17^. In such cases, the tooth color matching of single-shade composites relies on their ability to achieve a satisfactory color by mirroring the underlying dental substrate ^18^. This ability to recover the unprepared teeth's color could determine the suitability of simplified composite systems for large restorations. Unfortunately, there is a lack of information available regarding this matter.

This study aimed to assess the effectiveness of single-shade composites and a universal composite in restoring large cavities while achieving a similar color to unprepared teeth. Additionally, the study examined the color match between the composites and the surrounding tooth substrate, which was visually assessed by laypersons and undergraduate students of dentistry. The study hypothesized the following: 1) The evaluated composites cannot fully restore the original tooth color; 2) there is no significant difference among the composites concerning their ability to recover the tooth color; 3) the composites do not differ significantly in their ability to adjust the color to match the surrounding enamel, and 4) there is no difference in scores attributed to color matching between laypersons and undergraduate students of dentistry.

## Material and methods

### Experimental Design

In this in vitro study, we investigated the influence of different composite brands on tooth color. The independent variable, composite brand, was analyzed at four levels, including three single-shade composites: Palfique Omnichroma (Tokuyama Dental, Tokyo, Japan), Charisma Diamond One (Heraeus Kulzer, Hanau, Germany), and Vittra APS Unique (FGM, Joinville, SC, Brazil). The fourth level was the universal composite Filtek Universal (3M Oral Care, St. Paul, MN, USA).

The study focused on two dependent variables: the measured color differences between the restoration and the tooth surface before cavity preparation, which we assessed using the whitening index for dentistry (WI_D_), ΔE_ab,_ and ΔE_00_. Additionally, we conducted a visual analysis to evaluate the color adaptation of the restoration to the surrounding dental substrate.

Baseline Measurements and Specimen Allocation

Ten sound, fresh third molars were used for the study. The study protocol received approval from the scientific review committee and the committee for protecting human study participants at the local university (protocol CAAE 61770622.9.0000.5546). The roots of these molars were removed, and a section was made in the distal-mesial direction to separate the buccal and palatine surfaces. A clinical spectrophotometer (Easyshade Compact Advance 5.0, Vita-Zahnfabrik, Bad Säckingen, Germany) was employed to measure the color of these surfaces.

Before taking measurements, repositioning guides were created with silicone impression material to ensure consistent color measurement areas throughout the experiment. A 6-mm-diameter perforation was made on the guide to match the diameter of the spectrophotometer tip. The color coordinates L* (brightness), a* (hue in the red-green axis), and b* (hue in the blue-yellow axis) were recorded. The WI_D_ was calculated using Equation 1 ^20^:

Equation 1 :

$${WI}_{D}=0.551\times L^{*}\;-2.324\;\times a^{*}-1.1\times b^{*}$$

The twenty specimens (each representing half of the tooth crown) were ranked based on their WI_D_ values and subsequently divided into five blocks of four specimens each, ensuring that each block contained specimens with similar WI_D_ values. A single cavity was prepared on each specimen, and, within each block, the specimens were then randomly assigned to be restored with one of the evaluated composites. The random allocation was determined using a list generated on the website www.sealedenvelop.com.

### Cavity Preparation

For cavity preparation, repositioning guides were positioned over the dental surfaces. A cavity delimitation was made with a marking pen on the surface to indicate the diameter of the perforation. This delimitation served as a guide during the cavity preparation process. Cavity preparation was carried out using a coarse cylindrical diamond bur with a flat ending (#1090, KG Sorensen, Barueri, SP, Brazil) operated with a high-speed handpiece, and a copious air-water spray was used during the procedure. The final dimensions of the cavities were 6 mm in diameter and 2 mm in depth, and these measurements were verified using a digital caliper. The cavity margins were beveled with a conic diamond bur (1190F, KG Sorensen, Barueri, SP, Brazil).

### Restorative Procedures

The shade of the composite Filtek Universal was determined by placing small increments of available shades on the enamel surface. The increments were light-cured, and the shade that best matched the tooth color was selected. Specifically, shade A3 was chosen for four specimens, while shade A2 was used for the remaining specimen allocated for restoration with this composite.

For all composites, the enamel surrounding the cavities was etched using 37% phosphoric acid for 30 seconds. Afterward, the acid was removed with an air-water spray, and the dental substrates were dried with an airstream. The universal adhesive Ambar (FGM, Joinvile, SC, Brazil) was then actively applied onto the cavity walls and light-cured for 25 seconds. Subsequently, the composites were inserted into the cavities in a single increment and light-cured for 45 seconds. Finally, the restorations were polished using diamond discs (Sof-Lex, 3M Oral Care, St. Paul, MN, USA) in decreasing order of granulation, ranging from coarse to fine. Before conducting color assessments, the specimens were immersed in water and stored for one week.

### Color Recovery Analysis

The spectrophotometer tip was carefully positioned over the restorations to measure the color coordinates, using the repositioning guides as reference points. The WI_D_ values were then calculated based on the previously described equation 1. Moreover, the value difference between the restored and unprepared areas within the same specimen was determined. Additionally, the overall color difference between the restored and unprepared specimens was calculated using the CIE76 (equation 2) and CIEDE2000 formulas (>equation 3) ^21^ ^,^ ^22^.

Equation 2 :

$${∆E}_{ab}\;=\;{{{\left[(\Delta L^{*}\right)}^{2}+{\left(\Delta a^{*}\right)}^{2}+(\Delta b^{*})}^{2}]}^{1/2}$$

Equation 3 :

$$∆E_{00}=\;\sqrt{{\left(\frac{∆L^{'}}{K_{L}S_{L}}\right)}^{2}+{{\left(\frac{∆C^{'}}{K_{C}S_{C}}\right)}^{2}+\;\left(\frac{∆H^{'}}{K_{H}S_{H}}\right)}^{2}+\;R_{T}\frac{∆C^{'}}{K_{C}S_{C}}\;\frac{∆H^{'}}{K_{H}S_{H}}}$$

Where ΔL', ΔC', and ΔH' represent the changes in luminosity, chroma, and hue, respectively. SL, SC, and SH are the weighted functions for each component. KL, KC, and KH are the weighted factors for Lightness, Chroma, and Hue, respectively (KL = KC = KH = 1). RT is the interactive term between chroma and hue differences.

### Color Matching Analysis

To assess the color matching of the restorations to the surrounding tooth enamel, twenty evaluators participated in the study. This group comprised 20 laypersons with no prior experience in tooth color assessment and 20 undergraduate students of dentistry. Only final-year dental students with prior experience in restorative procedures involving color participated in the study. Before the evaluation, the evaluators' competence for color discrimination was tested using the Ishihara color plates. Any evaluators showing color deficiencies were replaced to ensure reliable assessments. All evaluators willingly participated and provided their informed consent by signing a participation agreement.

The experiments took place in a viewing booth, where the specimens were positioned on a neutral gray sample holder inclined at 45° to ensure consistent lighting from a D65 illuminant (CRI ≥ 90) ^23^. The viewing booth was in a completely dark room to facilitate visual adaptation to darkness before the assessments. To eliminate bias, the order of specimen evaluation was randomized for each evaluator using a random list generated on the website www.sealedenvelope.com. During the evaluation, the evaluators were instructed to compare the color of the restorations with the surrounding enamel and assign a score based on the color match. The scoring system was as follows: 0: Perfect match/No difference in color; 1: Close match/Small difference; 2: Good match/Acceptable; 3: Poor match/Hardly acceptable; and 4: Mismatch/Not acceptable.

### Data Analysis

The normal distribution of data was verified using the Shapiro-Wilk test, and the homogeneity of variance was assessed using Levene's test. Data of WID were analyzed by 2-way repeated measures (RM) ANOVA, analyzing the factors "composite" and "restoration (unprepared or restored tooth)," while this last was defined as a repetition factor. Data regarding ΔE_ab_, ΔE_00_, and changes in WI_D_ (ΔWI_D_) values were submitted to One-way ANOVA.

Regarding the visual analyses, we calculated the average of scores attributed by each evaluator for the five restorations using the same composite. Then, data were analyzed by 2-way RM ANOVA, assessing the factors "evaluator" (layperson vs. undergraduate student) and "composite." Pairwise comparisons were made using Tukey's test. A significance level of 95% was set for all data analyses.

## Results

### Color Recovery Analysis

The results of the instrumental analyses are shown in Table 1. For WI_D_, 2-way RM ANOVA showed that only the factor "restoration" (p < 0.001) affected the results. The factor "composite" alone (p = 0.613) did not intervene on WI_D_, but the interaction between the factors was significant (p = 0.017). Except for Filtek Universal, the restored teeth were whiter than the unprepared. Restorations built with Vittra Unique were whiter than those made using Filtek Universal. Similar findings were observed for ΔWI_D_ (p = 0.048), ΔE_ab_ (p = 0.045), and ΔE_00_ (p = 0.025).

**Table 1:** Means (standard deviation) for color differences between the unprepared and restored teeth (n = 5).

Resin composites	Whiteness index			ΔE_ab_	ΔE_00_
	Unprepared tooth	Restored tooth	Difference		
Omnichroma	16.3 (6.5) ^Ab^	32.2 (9.0) ^ABa^	15.8 (8.8) ^AB^	12.5 (3.9) ^B^	7.2 (2.1) ^B^
Vittra Unique	15.9 (6.0) ^Ab^	41.9 (1.9) ^Aa^	26.0 (6.7) ^A^	19.9 (4.7) ^A^	11.6 (2.1) ^A^
Charisma Diamond One	16.2 (7.7) ^Ab^	34.9 (8.5) ^ABa^	18.7 (5.8) ^AB^	17.1 (3.2) ^AB^	9.3 (1.8) ^AB^
Filtek Universal	19.3 (10.7) ^Aa^	27.9 (8.5) ^Ba^	8.6 (8.4) ^B^	10.8 (4.3) ^B^	6.2 (2.3) ^B^

For each outcome, distinct letters (uppercase comparing the rows, lower the lines) indicate statistical difference at Tukey`s test (p <0.05).

### Color Matching Analysis

Table 2 presents the results of the visual analysis. Two-way RM ANOVA showed that neither the "evaluator" (p = 0.104) nor the "composite" (p = 0.050) affected the scores attributed to the color adaptation of resin composite to adjacent tooth substrate. Still, the factors' interaction was also insignificant (p = 0.405). In general, undergraduate students of dentistry attributed higher scores (poorer adaptation) than laypersons. Regarding the resin composites, the highest scores were observed for the Filtek Universal composite, with statistical differences from Omnichroma and Vittra Unique.

**Table 2:** Means (standard deviation) of scores attributed by the evaluators regarding the color adjustment of the composite to the adjacent tooth structure.

Resin composites	Evaluators		Pooled average
	Layperson (n = 20)	Undergraduate student* (n = 20)	
Omnichroma	1.13 (0.59)	1.59 (0.60)	1.37 (0.63)
Vittra Unique	1.16 (0.71)	1.50 (0.63)	1.34 (0.68)
Charisma Diamond One	1.26 (0.75)	1.64 (0.61)	1.46 (0.70)
Filtek Universal	1.48 (0.76)	1.64 (0.51)	1.56 (0.63)
Pooled average	1.29 (0.70)	1.58 (0.59)	

For pooled averages, distinct letters indicate statistical differences at Tukey`s test (p <0.05).

Scores: 0: Perfect match/No difference in color; 1: Close match/Small difference; 2: Good match/Acceptable; 3: Poor match/Hardly acceptable; and 4: Mismatch/Not acceptable.

## Discussion

The results of this study revealed important findings about the different composites used for tooth restorations. Specifically, restorations with single-shade composites were noticeably whiter than the original teeth color. However, the universal composite restoration did not show a statistically significant difference in WI_D_ values compared to unprepared teeth. Notably, the Filtek Universal composite demonstrated the least change in WI_D,_ ΔE_ab_, and ΔE_00_ values, significantly outperforming the Vittra Unique composite. Consequently, the initial two hypotheses of the study cannot be accepted. On the other hand, there was no observable difference among the evaluated composites concerning their ability to adapt to the surrounding tooth substrate, which led to the non-rejection of the third hypothesis.

In studies assessing how well restorative materials adapt to dental substrates in terms of color, standardizing the substrate color is a challenge because it can influence the results. In our study, we addressed this issue by using the color of unprepared teeth not only to gauge the evaluated composites' ability to recover the tooth color but also to ensure a balance of teeth with similar colors among the materials. It has been demonstrated that single-shade composites tend to adjust better to whiter substrates ^14^ ^,^ ^15^ ^,^ ^16^. Therefore, if we had unbalanced the teeth color among the composites, it could have introduced bias by favoring the composite used in whiter teeth. The four darkest teeth placed in the same block had their WI_D_ values ranging from 6.0 to 9.9, while in the block with the whitest teeth, the values varied from 26.2 to 35.6. By doing this, we believe that our method minimized potential biases associated with substrate color and improved the reliability of our results.

The ability of single-shade composites to replicate the natural tooth color of surrounding substrates depends on their enhanced translucency compared to multi-shaded composite systems.^14^ ^,^ ^18^ This property allows these materials to be influenced significantly by the color of the underlying dental substrate, resulting in a "mirroring effect" on the final restoration color.^18^ It would be logical to assume that using a single-shade composite to fill cavities in sound teeth would lead to restorations with a color like the original tooth color before the cavity preparation. In such cases, the composite mainly replaces tooth enamel and is applied to sound, non-stained dentin. However, unexpectedly, the simplified restorative materials were unable to fully restore the original tooth color. A plausible explanation for this outcome is the relatively thick layer of composite (approximately 2 mm), which substantially reduces their ability to mirror the underlying substrate ^15^. As a result, the final restoration color becomes strongly influenced by the actual color of the composite, which is typically whiter than the natural tooth color ^14^ ^,^ ^15^ ^,^ ^16^.

On the other hand, the universal composites offered a variety of shades, and their translucency played a crucial role in achieving a color match to the natural tooth. By carefully selecting the composite shade that best matched the original tooth color, the likelihood of achieving a similar color in the restoration increased. Apart from one specimen (A2), the shade A3 proved to be the closest match to the teeth colors included in the study. Notably, A3 is a common tooth color available in the VITA classical shade guide ^24^, further reinforcing the findings observed for the single-shade composites, which showed less adaptability to darker substrates ^14^ ^,^ ^15^ ^,^ ^16^. It is important to emphasize that, even though there was no statistical difference, there was a clinically noticeable difference in WI_D_ values (8.6) between unprepared teeth and those restored with Filtek Universal. This difference is more than three times the value considered clinically unacceptable (2.6) in a prior study ^25^. Therefore, it is reasonable to expect that increasing the sample size would likely result in a statistically significant difference, which presents a limitation of the present study.

Although the universal composite performed better in terms of color recovery, all the evaluated materials demonstrated similar color adaptation to the enamel surrounding the restoration. This color adjustment ability is also influenced by the material's translucency, which enables the restoration color to blend seamlessly with the surrounding substrate. The presence of beveled margins further enhances this optical property, resulting in a less noticeable color transition. ^(^ ^26^ ^)^ In fact, the average scores for all evaluated composites fell between 1.34 and 1.56, indicating a good to very good color adaptation. These findings demonstrate that while both single-shade and universal composites have limited ability to fully recover the original tooth color, they do exhibit adequate color adaptation to the tooth substrate surrounding the restoration. As a result, restorations can be achieved with at least a good color match to the natural teeth.

Despite the satisfactory color match to the surrounding tooth substrate, large restorations made with single-shade composites were noticeably whiter than the original, unprepared teeth color. Even with the universal composite, which showed reduced color discrepancy due to prior shade selection, the material still couldn't fully recover the natural color of the unprepared teeth. These findings demonstrate that using this simplified composite system may not achieve a similar color to an adjacent tooth when dealing with large restorations, even if the underlying tooth substrate is sound. In such cases, it appears that stratifying the restorations using composites with multiple shades and varying translucency remains a better option for achieving more aesthetic results. It's essential to note that the behavior of composites can differ based on different cavity configurations, especially when using thinner layers. Therefore, further studies are necessary to better understand these simplified composite systems and their performance in various scenarios.
